# Residence- and parental education-related social inequalities in early childhood caries: cross-sectional national Lithuanian oral health survey

**DOI:** 10.1186/s12903-026-09311-5

**Published:** 2026-07-18

**Authors:** Gabriele Simenaite, Indre Stankeviciene, Vilma Brukiene, Alina Puriene, Agne Roviene, Jolanta Aleksejuniene, Lina Stangvaltaite-Mouhat

**Affiliations:** 1 Institute of Dentistry, Faculty of Medicine, Vilnius University, Vilnius, Lithuania; 2 Private Practice, Vilnius, Lithuania; 3 Department of Oral Health Sciences, Faculty of Dentistry, University of British Columbia, Vancouver, Canada; 4 Oral Health Centre of Expertise in Eastern Norway, Oslo, Norway

**Keywords:** Pediatric Dentistry, Dentistry, Oral Health, Dental Care for Children, Social Determinants of Health

## Abstract

**Background:**

Early childhood caries (ECC) is a highly prevalent public health problem impacting children under the age of six. The study assessed the prevalence and experience of ECC among 3- and 5-year-olds in Lithuania and investigated residence- and parental education-related social inequalities.

**Methods:**

This cross-sectional study used the National Lithuanian Oral Health Survey (2017–2019) data. Parents and their 3- and 5-year-olds attending kindergartens from the five largest Lithuania’s cities and randomly selected 10 peri-urban/rural areas were invited to participate. Children’s caries experience (decayed, missing, filled teeth [d_3_mft]; [d_3_t]; [mt]; [ft] indices) and oral hygiene status (Silness-Löe index) were recorded. The World Health Organization Oral Health Questionnaire for Adults was used to collect data about family’s residence (urban, peri-urban, rural) and parental education (secondary or lower, college, university). Information regarding fluoride concentration in community water was retrieved directly from the water suppliers. The analytical strategy was based on the WHO conceptual framework for action on the social determinants of health. Negative binomial regression analyses were used.

**Results:**

In total, 445 3- and 5-year-olds (227, 51.0% girls) participated in the study. The prevalence of ECC among the 3-year-olds and 5-year-olds was 30.9% and 66.9%, respectively. Mean (SD) d_3_mft score was 2.0 (3.6) among the 3-year-olds and 3.4 (3.7) among the 5-year-olds. Compared with urban residence, rural and peri-urban residence were associated with higher mean d_3_mft (RM 1.73, 95% CI 1.22–2.45; and RM 1.60, 95% CI 1.21–2.13, respectively) and higher mean d_3_t scores (RM 2.15, 95% CI 1.50–3.08; and RM 1.63, 95% CI 1.20–2.21, respectively). Mean mt scores were approximately 2.7 times higher of children whose parents had secondary or lower (RM 2.71, CI 95% 1.32–5.58) or college (RM 2.90, CI 95% 1.47–5.74) education as compared to the ones with university education. Analyses stratified by parental education indicated that higher ECC experience was observed in 3- and 5-year-olds whose parents had secondary or lower education and resided in rural or peri-urban areas.

**Conclusions:**

The prevalence and experience of ECC among 3- and 5-year-olds was high. Secondary or lower parental education and residence in rural or peri-urban areas resulted in higher rates of overall ECC experience and untreated caries. Parental education-related inequalities were observed in treatment experience. The study suggests the need for the implementation of oral health-promoting programs targeting preschoolers in Lithuania with focus on reducing inequalities.

**Supplementary Information:**

The online version contains supplementary material available at https://doi.org/10.1186/s12903-026-09311-5.

## Background

Oral diseases are a major public health problem, impacting 514 million children worldwide [1]. The term “early childhood caries” (ECC) was specifically referred to the paediatric population and redefined in the 2018 Bangkok Declaration as the presence of one or more decayed (cavitated or non-cavitated), missing or filled (due to caries) primary teeth in children under the age of six [2]. The global pooled prevalence of ECC is estimated to be 48% [3]. Untreated dental caries negatively impacts child’s physical and mental health [3]. Pain, eating and sleeping difficulties, aesthetic and social concerns are all attributable to ECC [4, 5]. Untreated caries lesions can result in severe infections requiring acute hospital admission and treatment under general anesthesia causing a major financial burden for the individuals and health care systems [6, 7].

Dental caries is a complex, multifactorial, biofilm-mediated disease damaging the dental tissues [8, 9]. Manifestation of tooth decay in children is determined by biological, dietary, behavioural and psychosocial aspects [10]. According to the World Health Organization’s (WHO) conceptual framework for action on the social determinants of health [11], structural factors, for example, socioeconomic position of parents, shape the above-mentioned intermediary determinants of oral health. Indicators of underprivileged socioeconomic position such as lower parental education are commonly associated with higher ECC prevalence in the preschool age group [10, 12, 13].

Historically, prevalence of ECC in Lithuanian children has been extremely high. During the early 2000s, 50% of children aged 3 years and almost 90% of 4-6-year-olds suffered from ECC [14, 15]. An improvement regarding the ECC during the last decade was observed, however, further progress is still needed [16]. Existing research demonstrated residence-related inequalities in dental caries experience among Lithuanian adults [17]. Social inequalities in oral health were examined in a previous adolescent Lithuanian study where higher family affluence was associated with higher oral health-related quality of life [18]. A study investigating the preschoolers’ oral health reported that parents with higher education levels and parents residing in urban areas exhibited more positive attitudes towards oral health care [16]. High caries intensity was also observed in 7-8-year-olds from rural areas [19]. Previous Lithuanian studies reported social inequalities in oral health in various age groups. However, these reports did not investigate ECC as a primary focus. Therefore, the aim of the current study was to assess ECC prevalence and experience as well as examine residence- and parental education-related social inequalities in 3- and 5-year-old Lithuanians. The null hypothesis stated that residence and parental education were not associated with ECC experience.

## Methods

### Study design and sample size

The 2017–2019 National Lithuanian Oral Health Survey (NLOHS) invited 3- and 5-year-olds and one of their parents to participate in a cross-sectional epidemiological study. Two key age groups of 3- and 5-year-olds were chosen to enable a comparison with other studies, and this choice was also in accordance with WHO recommendations for population surveys [15, 20]. Following the WHO Oral Health Survey Basic Methods recommendations, a multi-cluster random sampling framework was chosen. Five largest Lithuania’s cities and randomly selected 10 peri-urban areas, one from each of the 10 counties, were included. Subsequently, randomly selected kindergartens from each area were selected to recruit the study participants [20]. Criteria for inclusion were 3 and 5 years of age and provision of informed consent by parents or guardians. Children whose parents or guardians were not able or refused to give informed consent were not included in the study. The presence of medical conditions (systemic diseases or physical disabilities) was not regarded as exclusion criteria.

Sample size calculation was based on a previous study showing that the prevalence of ECC in 3-year-olds was 50% [15], estimating the expected proportion with 5% absolute precision and 90% confidence. The calculated minimum number of 271 participants due to the multi-cluster random sampling was multiplied by a design effect that was arbitrarily set at 1.5 [21] which resulted in minimum recruitment of 406 participants for each age group.

### Clinical examination

One trained and calibrated examiner (AR, Intraclass Correlation Coefficient = 0.95 for d_3_mft evaluation based on duplicate recordings of ten patients) performed children’s oral examination according to the WHO Oral Health Survey Basic Methods recommendations [20]. Prior to the examinations, parents or guardians of children in each selected kindergarten were informed about the study and examination process. All 3- and 5-year-olds who were present in kindergarten on the day of examination and whose parents or guardians provided informed consent were examined. Examinations took place at kindergartens with participants seated in a chair. A plane mouth mirror (Hu-Friedy, USA), Community Periodontal Index probe (Hu-Friedy, USA) and a portable examination light were used. Caries experience was measured as individual total numbers of decayed teeth (d_3_t), missing teeth (mt), filled teeth (ft) and decayed, missing, filled teeth (d_3_mft). Dental caries was considered to be present when the dentin lesions were involved [20]. Teeth missing due to physiological exfoliation were excluded from the count of missing teeth. Oral hygiene was evaluated according to the Silness-Löe index scoring as good (0.1-1.0), moderate (1.1-2.0) or poor (2.1-3.0) [22].

### Other variables

One of the parents filled in WHO Oral Health Questionnaire for Adults [20]. Information about family’s place of residence (urban, peri-urban, rural [20]), parental education (secondary or lower, college, university [23]), and child’s sex (male, female) were recorded. Fluoride levels in community water were retrieved directly from the water providers in accordance with participants‘ residence, and subsequently dichotomized into < 0.7 mg/L and ≥ 0.7 mg/L categories [24].

### Statistical analysis

**Fig. 1:**
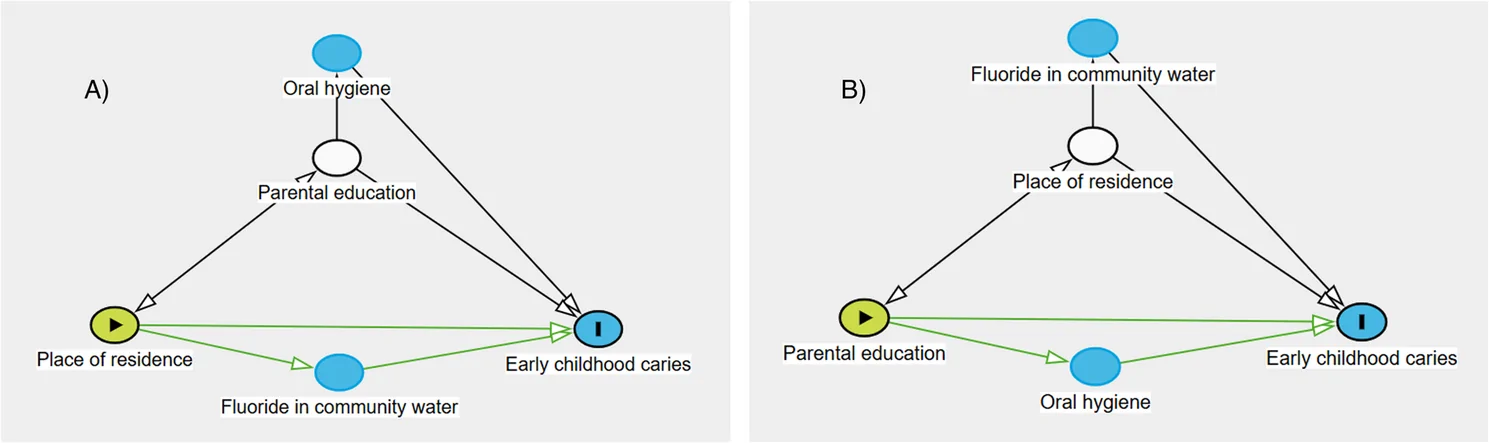
A simplified directed acyclic graph (DAG). DAG was used to guide the selection of covariates for adjustment in the analysis: (**A**) place of residence (independent variable) and ECC (outcome) with adjusted parental education as potential confounding factor (in white), and unadjusted fluoride levels in community water and oral hygiene as potential mediating factors (in blue); (**B**) parental education (independent variable) and ECC (outcome) with adjusted place of residence as potential confounding factor (in white), and unadjusted fluoride levels in community water and oral hygiene as potential mediating factors (in blue)

For statistical analysis, IBM SPSS Statistics (version 27) software was used (IBM, Armonk, NY, USA) and forest plots were generated using R Statistical Software (version 4.5.1, R Foundation for Statistical Computing, Vienna, Austria) with relevant packages in RStudio (version 2025.09.1 + 401, Posit Software, PBC, Boston, MA, USA). Mann-Whitney U and Kruskal-Wallis tests were used. The analytical strategy and the assumed relationship between variables used in the analyses were based on the WHO conceptual framework for action on the social determinants of health [11] and illustrated using a simplified directed acyclic graph (DAG) (Fig. 1). DAG was used to guide the selection of covariates for adjustment in the analysis: (A) Place of residence (independent variable) and ECC (outcome) with adjusted parental education as potential confounding factor (in white), and unadjusted fluoride levels in community water and oral hygiene as potential mediating factors (in blue); (B) Parental education (independent variable) and ECC (outcome) with adjusted place of residence as a potential confounding factor (in white), and unadjusted fluoride levels in community water and oral hygiene as potential mediating factors (in blue). To examine associations between the place of residence, parental education and ECC experience, negative binomial regression analyses were employed, as the overdispersion was present in the count outcome variables (d_3_mft, d_3_t, mt, ft). The interaction between parental education and place of residence was explored and resulted in statistical significance (*p* < 0.001). Subsequently, the subgroup analyses stratified by parental education were conducted. Stratified analysis for mt as outcome was not performed due to low event count per group. Threshold for statistical significance was set at *p*≤0.05, ratio of means (RM) and their 95% confidence intervals (CI) were presented. Cases with missing data were excluded from the analysis. Sensitivity analyses adjusted for all covariates were performed (see Additional file 1).

The clustering effect was not statistically significant when kindergartens were used as clusters, therefore multilevel analyses were not performed.

The post-hoc power calculation indicated 100% statistical power when using mean caries experience (d_3_mft) as an outcome measure based on estimates from a previous study reporting a mean caries experience (dmft) of 2.1 (standard deviation [SD] 0.1) [15].

This study was reported in accordance with the Strengthening the Reporting of Observational Studies in Epidemiology (STROBE) recommendations (see Additional file 2).

## Results

In total, 445 children (*n* = 227, 51.0% girls), out of which 257 (57.8%) were 5-year-olds and 188 (42.2%) were 3-year-olds participated in the study (Tables 1 and 2).

**Table 1:** Comparison of the d_3_mft, d_3_t, mt and ft scores of 3-year-olds (*n* = 188) according to the caries determinants

Total		d_3_mft (decayed, missing, filled teeth)		d_3_t (decayed teeth)		mt (missing teeth)		ft (filled teeth)	
	*n* (%)	Mean (SD)	*P* values	Mean (SD)	*P* values	Mean (SD)	*P* values	Mean (SD)	*P* values
	188 (100.0)	2.0 (3.6)	*-*	1.4 (3.0)	*-*	0.1 (0.8)	*-*	0.5 (1.4)	*-*
DETERMINANTS									
Place of residence^1^									
Urban	113 (60.1)	1.7 (3.1)	0.149	1.1 (2.3)	0.343	0.1 (0.9)	0.552	0.5 (1.4)	0.550
Peri-urban	47 (25.0)	2.3 (4.2)		1.8 (4.0)		0.2 (0.7)		0.4 (1.1)	
Rural	28 (14.9)	3.1 (4.2)		2.2 (3.3)		0.1 (0.4)		0.8 (1.8)	
Parental education^1^									
Secondary or lower	49 (26.1)	2.2 (4.1)	0.075	1.7 (3.9)	0.114	0.1 (0.4)	0.561	0.4 (1.2)	0.068
College	48 (25.5)	2.7 (4.0)		1.9 (3.1)		0.3 (1.2)		0.6 (1.2)	
University	81 (43.1)	1.8 (3.1)		1.1 (2.4)		0.1 (0.6)		0.6 (1.6)	
Sex^2^									
Male	92 (48.9)	1.5 (2.8)	0.339	0.9 (2.1)	0.681	0.2 (1.0)	0.223	0.4 (1.0)	0.555
Female	96 (51.1)	2.6 (4.2)		1.9 (3.6)		0.1 (0.5)		0.6 (1.7)	
Fluoride level in community water^2^									
< 0.7 mg/L	132 (70.2)	2.4 (3.9)	0.137	1.7 (3.3)	0.165	0.2 (0.9)	0.364	0.6 (1.5)	0.531
≥ 0.7 mg/L	56 (29.8)	1.2 (2.4)		0.8 (1.9)		0.1 (0.5)		0.4 (1.0)	
Oral hygiene^1^									
Good	123 (65.4)	1.5 (3.1)	< 0.001	1.0 (2.6)	< 0.001	0.2 (0.9)	0.822	0.3 (1.2)	0.002
Moderate	56 (29.8)	2.6 (3.9)		1.7 (3.0)		0.1 (0.6)		0.8 (1.6)	
Poor	9 (4.8)	5.8 (5.6)		4.7 (5.7)		0.0 (0.0)		1.1 (1.7)	

^1^ Kruskal-Wallis test; ^2^ Mann-Whitney U test

**Table 2:** Comparison of the d_3_mft, d_3_t, mt and ft scores of 5-year-olds (*n* = 257) according to the caries determinants

Total		d_3_mft (decayed, missing, filled teeth)		d_3_t (decayed teeth)		mt (missing teeth)		ft (filled teeth)	
	*n* (%)	Mean (SD)	*P* values	Mean (SD)	*P* values	Mean (SD)	*P* values	Mean (SD)	*P* values
	257 (100.0)	3.4 (3.7)	*-*	2.0 (2.9)	*-*	0.2 (0.7)	*-*	1.3 (1.8)	*-*
DETERMINANTS									
Place of residence^1^									
Urban	155 (60.3)	2.7 (3.2)	0.001	1.3 (2.1)	< 0.001	0.1 (0.5)	0.423	1.2 (1.7)	0.085
Peri-urban	70 (27.2)	4.6 (4.0)		2.8 (3.4)		0.2 (1.0)		1.6 (2.1)	
Rural	32 (12.5)	4.4 (4.4)		3.5 (4.1)		0.2 (0.8)		0.7 (1.3)	
Parental education^1^									
Secondary or lower	64 (24.9)	4.3 (4.2)	0.004	3.2 (3.9)	0.002	0.3 (1.2)	0.146	0.8 (1.5)	0.003
College	57 (22.2)	3.8 (3.4)		1.9 (2.5)		0.2 (0.8)		1.8 (1.9)	
University	132 (51.4)	2.6 (3.4)		1.3 (2.2)		0.1 (0.3)		1.2 (1.9)	
Sex^2^									
Male	126 (49.0)	3.7 (3.6)	0.232	2.2 (2.9)	0.158	0.1 (0.6)	0.865	1.3 (1.9)	0.903
Female	131 (51.0)	3.2 (3.9)		1.8 (3.0)		0.2 (0.8)		1.2 (1.8)	
Fluoride in community water^2^									
< 0.7 mg/L	161 (62.6)	3.9 (3.8)	0.008	2.3 (3.2)	0.123	0.2 (0.8)	0.496	1.4 (1.9)	0.039
≥ 0.7 mg/L	96 (37.4)	2.7 (3.4)		1.6 (2.4)		0.1 (0.6)		1.0 (1.7)	
Oral hygiene^1^									
Good	155 (60.3)	2.4 (2.9)	< 0.001	1.1 (1.8)	< 0.001	0.2 (0.8)	0.013	1.1 (1.6)	0.508
Moderate	84 (31.7)	4.5 (4.3)		3.0 (3.6)		0.1 (0.3)		1.4 (2.1)	
Poor	18 (7.0)	7.6 (3.1)		5.2 (3.5)		0.6 (1.3)		1.7 (2.1)	

^1^ Kruskal-Wallis test; ^2^ Mann-Whitney U test

The prevalence of ECC among 3-year-olds was 30.9% and among 5-year-olds it was 66.9%. The mean d_3_mft score of the 3-year-olds was 2.0 (SD 3.6) with the median of 0.0 (interquartile range [IQR] 2.0). The mean d_3_mft score of the 5-year-olds was 3.4 (SD 3.7) with the median of 2.0 (IQR 6.0) (Tables 1 and 2).

In 3-year-olds, differences in the d₃mft score and its individual components, except mt, were observed across the oral hygiene groups (Table 1).

In 5-year-olds, mean differences in the d₃mft scores were observed across groups of children with variations in their residence, parental education, fluoride level in community water, and oral hygiene (Table 2).

A gradient across different residence categories was observed for d_3_mft and d_3_t scores. Children residing in rural areas had, on average, a 73% higher d_3_mft scores than those living in urban areas (RM 1.73, 95% CI 1.22–2.45) while children from peri-urban areas had, on average, a 60% higher d_3_mft scores than those living in urban areas (RM 1.60, 95% CI 1.21–2.13). Compared to urban residents, rural residents had 2.15 higher mean d_3_t score (RM 2.15, 95% CI 1.50–3.08) and mean d_3_t score of peri-urban ones was 63% greater (RM 1.63, 95% CI 1.20–2.21) (Table 3).

**Table 3:** Multivariable negative binomial regression analyses of the association between residence, parental education and ECC experience (d_3_mft index and its individual components d_3_t, mt, ft) (*n* = 431)

DETERMINANTS^	d_3_mft (decayed, missing, filled teeth)		d_3_t (decayed teeth)		mt (missing teeth)		ft (filled teeth)	
	RM (95% CI)	*P* values	RM (95% CI)	*P* values	RM (95% CI)	*P* values	RM (95% CI)	*P* values
Place of residence (ref. Urban)								
Peri-urban	1.60 (1.21; 2.13)	0.001	1.63 (1.20; 2.21)	0.002	1.29 (0.69; 2.43)	0.422	1.54 (1.10; 2.14)	0.011
Rural	1.73 (1.22; 2.45)	0.002	2.15 (1.50; 3.08)	< 0.001	0.85 (0.36; 2.02)	0.707	1.06 (0.68; 1.65)	0.809
Parental education (ref. University)								
College	1.36 (1.03; 1.79)	0.028	1.40 (1.03; 1.89)	0.029	2.90 (1.47; 5.74)	0.002	1.19 (0.86; 1.64)	0.298
Secondary or lower	1.12 (0.83; 1.52)	0.449	1.51 (1.10; 2.08)	0.011	2.71 (1.32; 5.58)	0.007	0.56 (0.38; 0.82)	0.003

*ref.* Reference category, *RM * Ratio of means, *CI * Confidence interval

^Multicollinearity diagnostics revealed no multicollinearity among determinants (VIF<5)

Parental education-related inequalities in ECC experience were observed for d_3_t, mt and ft scores. Children whose parents had secondary or lower education, had, on average, 51% higher d_3_t score (RM 1.51, 95% CI 1.10–2.08) compared to parents who had university education. Mean d_3_t score of children whose parents had college education (in comparison to university education) were approximately 40% higher (RM 1.40, 95% CI 1.03–1.89). Mean mt scores were more than twice higher in children whose parents had secondary or lower (RM 2.71, CI 95% 1.32–5.58) or college (RM 2.90, CI 95% 1.47–5.74) education compared to children having parents with university education. Secondary or lower parental education was significantly associated with children’s lower mean ft score (RM 0.56, CI 95% 0.38–0.82) (Table 3).

Sensitivity analyses adjusted for all covariates generated estimates of a similar strength (see Additional file 1).

**Fig. 2:**
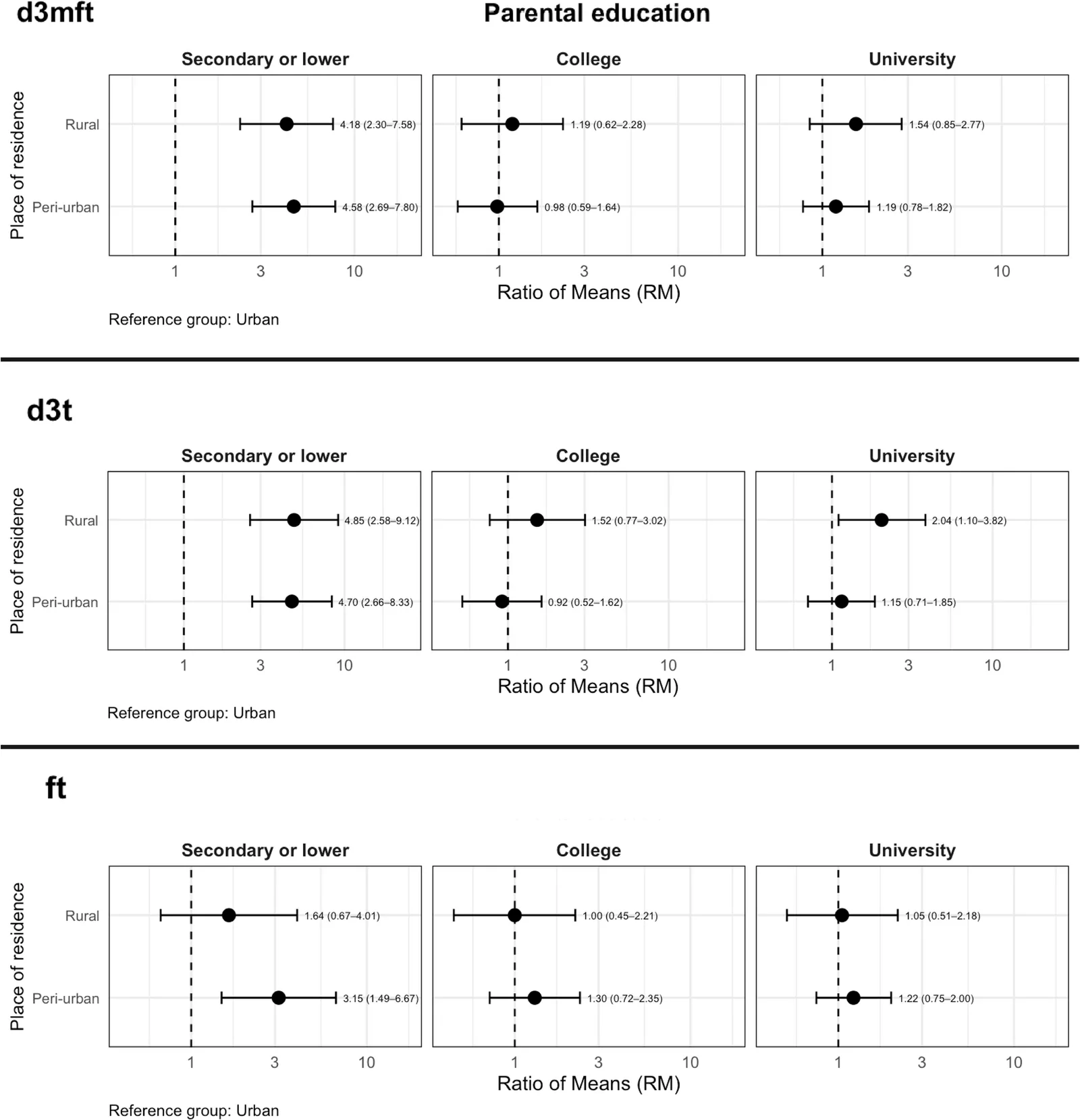
Forest plots reflecting ratio of means (RM) with 95% confidence intervals for d_3_mft, d_3_t and ft scores of 3- and 5-year-olds. Estimates compare rural and peri-urban place of residence with urban as a reference category. Analyses are stratified by parental education (secondary or lower, college, university)

According to analyses stratified by parental education, children of parents with secondary or lower education living in rural areas (versus urban) had significantly higher mean d_3_mft (RM 4.18, CI 95% 2.30–7.58) and d_3_t (RM 4.85, CI 95% 2.58–9.12) scores (Fig. 2). A similar pattern was observed in peri-urban areas, with significantly higher mean d_3_mft (RM 4.58, CI 95% 2.69–7.80) and d_3_t (RM 4.70, CI 95% 2.66–8.33) scores. Children of parents with university education and residing in rural areas had higher mean d_3_t score (RM 2.04, CI 95% 1.10–3.82).

The following variables had 0% missing data: d₃mft; d₃t; mt; ft; place of residence; sex of the child; fluoride in community water; oral hygiene. The proportion of missing data for parental education was 3.2%.

## Discussion

The present study demonstrated that every third 3-year-old and two thirds of 5-year-olds in Lithuania had ECC. Secondary or lower parental education and residence in rural or peri-urban areas resulted in higher rates of overall ECC experience and untreated caries. Parental education-related inequalities were observed in treatment experience. These findings confirmed the hypothesis of our study.

Even though the direct comparisons to previous studies due to differences in methodology cannot be made, it seems that ECC prevalence in Lithuania has decreased: from 50% among 3-year-olds in 2003 to 31% in 2017–2019 [15] and from 90.2% among 5-year-olds in 2010 to 67% in 2017–2019 [14]. No nationwide oral health prevention program targeting preschoolers in Lithuania has been implemented. The policy requiring a provision of a child’s health certificate to educational institutions, including documentation of a child’s oral health status was introduced in 2013 [25]. Thus, since 2013 parents have been obliged to take their child for an oral examination at least once a year starting at kindergarten age. It has been shown that the percentage of regular dental visits of preschool children increased from 36.4% in 2010/2011 to 85.1% in 2023 [16]. Additionally, there may have been a change in parental attitudes towards their children’s oral health. According to a recent Lithuanian repeated cross-sectional study on preschoolers, the proportion of parents who assisted their child’s toothbrushing increased from 19.2% to 85.1% in the 12-years observation period [16]. Despite relative improvements, meta-analysis on global ECC prevalence mentioned Lithuania as a country with the highest prevalence of ECC in the European region [3]. This finding was based on 2012 study results [14] which reported an 88.4% prevalence of ECC among 4-6-year-olds – slightly more than double the pooled ECC prevalence of 43% in the European region. The prevalence of ECC in Lithuania observed in the current study (54.4%) is now more in line with the reported European and global data [3].

ECC experience, as indicated by mean d_3_mft score, was 2.1 and 3.4 among 3- and 5-year-olds, respectively. A recent meta-analysis reported the global estimated random-effect pooled mean dmft of 3.7 in children under 71 months. The results of this study varied from the lowest mean score of 2.3 in Europe to the highest mean score of 5.5 in the Middle East region [26]. A 2018 systematic review on global ECC data among 5-year-olds reported the mean dmft to vary from 0.9 to 7.5 [27]. According to these reports, ECC experience in Lithuanian preschoolers appears to be moderate considering the global context. However, when focusing on Europe, lower mean dmft scores (0.2–1.9) were reported in several Western European countries such as Germany, Italy, France and United Kingdom [26, 27]. One of the main reasons for such country-based disparities might potentially be the influence of historical factors. In many post-Soviet countries, dental care rapidly transitioned to the private sector after the end of the Soviet era in the early 1990s [28]. Among the European countries, Lithuania experienced the highest growth of private dental expenditure in 2008–2019 [28]. Lithuania ranks among the top countries in the European Union where dental care expenses cause the highest financial burden on the population [29]. Free of charge dental treatment for children is provided in public clinics in Lithuania [30], however not all parents choose to use it. Additionally, preventive individual oral health behaviors were shown to be poorer in post-Soviet countries. A WHO report on adolescent health found that the lowest prevalence of toothbrushing was observed mostly in post-Soviet countries [31]. Similarly, a recent study reported poor oral hygiene habits among school-aged children from post-Soviet countries [32]. It can be argued that another significant factor associated with higher caries experience in post-Soviet countries is possibly the dominating treatment-oriented approach to health rather than the preventive one. In 2022, Germany and Austria had the highest preventive care expenditure per inhabitant while the lowest expenditures were observed in Eastern European countries such as Poland and Romania [33]. Lastly, socioeconomic position in Western Europe is overall higher which is indicated by higher income levels [34] or higher life expectancy [35]. Better oral health was associated with higher socioeconomic position, regardless of the age group studied [36].

In the present study, secondary or lower parental education and residence in rural or peri-urban areas resulted in higher rates of overall ECC experience and untreated caries. Higher number of untreated dental caries in children from rural areas were also reported in England, Wales and Northern Ireland (5-year-olds) [37], Poland (3-6-year-olds) [38], and Austria (6-12-year-olds) [39]. A scoping review on the association between ECC and Sustainable Development Goal 11 (to make cities and human settlements inclusive, safe, resilient and sustainable [40]) reported that links between urbanization and ECC are highly diverse. This review suggested that these differences may be associated with variations in country income levels, as no relationship between urbanization and ECC was observed in high-income countries [41]. In line with this study, a meta-analysis on global ECC data also reported a link between gross national income per capita (GNI) and ECC – higher GNI was associated with lower pooled mean caries experience [26]. This is quite a debatable finding since Lithuania has been classified as a high-income country by the World Bank since 2012 [42]. This discrepancy might be attributed to more complex patterns of social inequalities. Specific to Lithuania, individuals with higher educational levels, especially younger ones, are more likely to leave rural areas and move to urban areas for better further education and employment opportunities [43]. Moreover, residents in rural areas tend to have lower literacy levels and academic performance of rural students is persistently lower than that of urban students [44]. Lithuanian rural residents are also more likely to be unemployed long-term [45]. Therefore, rural households often may be of lower socioeconomic position, consequently more at risk of poverty [46, 47].

Additionally, the extent of public services in rural areas is limited due to shortage of healthcare workforce as well as long travel distances or waiting times [45, 48, 49]. Studies showed the association between impaired access to oral care services and worse oral health outcomes in children [48, 50, 51]. This may explain our finding of higher untreated caries rates among children in rural areas, even when their parents had university education. Strategies to address residence-related inequalities in ECC could be subsidizing transportation costs for patients and offering incentives to attract dental workforce to rural areas.

Parental education-related inequalities were also observed in 3- and 5-year-olds’ dental treatment experience. Children whose parents had lower education had significantly more missing teeth. This may be associated with several factors impacting children of less-educated parents: delayed dental visits leading to development of severe caries or other conditions where the impacted teeth of a child are no longer restorable, or preference for child‘s tooth extraction over restoration [7, 52, 53]. The first pathway reflects an unmet need for timely dental treatment which could be interpreted as children‘s dental neglect [54]. A systematic review reported that level of parental education was a significant risk factor for dental neglect [55]. Well-educated parents are more likely to possess greater knowledge on oral health, and their children potentially establish and maintain effective oral hygiene routines [56], have less cariogenic dietary habits and adopt preventive oral health practices [57]. Parents with higher level of education are also more likely to have more favourable employment positions and afford better oral health care for their children [58]. Therefore, it is essential to develop targeted interventions focused on preschoolers aimed at reducing inequalities related to the education of their parents. One of the effective ways to raise parental awareness regarding oral health might be the promotion of oral health in public places such as primary healthcare centers, nurseries and kindergartens [59]. Implementing food policy regulations that support healthier food choices for preschool children in these institutions may also lead to improvements in oral health outcomes. Introducing sugar tax is another potentially effective public health policy aimed at improving oral health of the preschool children. An umbrella review reported that a 20% volumetric tax to sugar-sweetened beverages could reduce caries occurrence by 2.7–2.9% in children over a 10-year period [60]. In Lithuania, an excise tax on sugar-sweetened non-alcoholic beverages is in effect since the beginning of 2026 [61].

One of our study’s limitations was a cross-sectional design which does not allow to establish causal inference. Moreover, the minimal calculated sample size for ECC prevalence was not reached. Only children who were present in kindergarten on the day of examination participated in the study. However, the outcome in the present study was ECC experience, and the post-hoc power calculation for ECC experience indicated 100% statistical power. Furthermore, the estimated association between rural/peri-urban place of residence and overall ECC experience, as well as untreated dental caries, is relatively precise, as reflected by narrow confidence intervals. In contrast, the confidence intervals for the estimated association between lower parental education and higher missing teeth count are wider, indicating greater uncertainty and less precise estimates. Due to low event count per group, we were unable to perform analysis stratified by parental education for mt score as an outcome. Another notable limitation was the use of a questionnaire, which relies on subjective self-reports of parental education and place of residence. In addition, we used previously collected data that were originally gathered for a different aim, and several important variables were not available, including key structural determinants of health such as household income. Due to similar context, findings of the study may be generalisable to other post-Soviet countries. However, the study results should be interpreted with caution given its limitations.

## Conclusions

The NLOHS showed that the prevalence and experience of ECC among 3- and 5-year-olds was high. Secondary or lower parental education and residence in rural or peri-urban areas resulted in higher rates of overall ECC experience and untreated caries. Parental education-related inequalities were observed in treatment experience. The study suggests the need for the implementation of oral health-promoting programs targeting preschoolers in Lithuania with focus on reducing inequalities.

## Supplementary Information

Additional file 1. Sensitivity analysis for multivariable negative binomial regression of the association between place of residence, parental education and early childhood caries experience (d3mft index and its individual components d3t, mt, ft) in participants (n=431)

Additional file 2. STROBE (Strengthening the Reporting of Observational Studies in Epidemiology) checklist. Page numbers indicate where items are reported in the manuscript

## Data Availability

No datasets were generated or analysed during the current study.

## Abbreviations

CI: Confidence interval
d_3_mft: Decayed, missing, filled teeth
d_3_t: Decayed teeth
ECC: Early childhood caries
ft: Filled teeth
GNI: Gross national income
IQR: Interquartile range
mt: Missing teeth
NLOHS: National Lithuanian Oral Health Survey
RM: Ratio of means
SD: Standard deviation
STROBE: Strengthening the Reporting of Observational Studies in Epidemiology
WHO: World Health Organization

## References

[1] World Health Organization. Global oral health status report: towards universal health coverage for oral health by 2030. 2022. https://www.who.int/publications/i/item/9789240061484. Accessed 10 Jan 2026.

[2] Pitts N, Baez R, Diaz-Guallory C, et al. Early Childhood Caries: IAPD Bangkok Declaration. Int J Paediatr Dent. 2019. 10.1111/ipd.12490. 31395110

[3] Uribe SE, Innes N, Maldupa I. The global prevalence of early childhood caries: A systematic review with meta-analysis using the WHO diagnostic criteria. Int J Paediatr Dent. 2021. 10.1111/ipd.12783. 33735529

[4] Foláyan MO, Ishola AG, Abodunrin OR, Ndembi N, El Tantawi M. Untreated early childhood caries is a potential disability: policy and programme implications for Africa. Front Oral Health. 2025. 10.3389/froh.2025.1546747. PMC12133737 40469389

[5] Pakkhesal M, Riyahi E, Naghavi Alhosseini A, Amdjadi P, Behnampour N. Impact of dental caries on oral health related quality of life among preschool children: perceptions of parents. BMC Oral Health. 2021. 10.1186/s12903-021-01396-4. PMC7885600 33588827

[6] Andrew L, Wallace R, Wickens N, Patel J. Early childhood caries, primary caregiver oral health knowledge and behaviours and associated sociological factors in Australia: a systematic scoping review. BMC Oral Health. 2021. 10.1186/s12903-021-01887-4. PMC8513214 34645446

[7] Rasteniene R, Simenaite G, Brukiene V. Maxillofacial infections in lithuanian hospitalised children and adolescents: a 17-years retrospective study. Eur Archives Pediatr Dentistry. 2023. 10.1007/s40368-023-00824-z. 37452905

[8] Tinanoff N, Baez RJ, Diaz Guillory C, Donly KJ, Feldens CA, McGrath C, et al. Early childhood caries epidemiology, aetiology, risk assessment, societal burden, management, education, and policy: Global perspective. Int J Paediatr Dent. 2019. 10.1111/ipd.12484. 31099128

[9] Moussa DG, Ahmad P, Mansour TA, Siqueira WL. Current State and Challenges of the Global Outcomes of Dental Caries Research in the Meta-Omics Era. Front Cell Infect Microbiol. 2022. 10.3389/fcimb.2022.887907. PMC9247192 35782115

[10] Anil S, Anand PS. Early Childhood Caries: Prevalence, Risk Factors, and Prevention. Front Pediatr. 2017. 10.3389/fped.2017.00157. PMC5514393 28770188

[11] Solar Orielle IA. A conceptual framework for action on the social determinants of health. Social Determinants of Health Discussion Paper 2 (Policy and Practice). 2010. https://www.afro.who.int/sites/default/files/2017-06/SDH_conceptual_framework_for_action.pdf. Accessed 24 Jan 2026.

[12] Lam PPY, Chua H, Ekambaram M, Lo ECM, Yiu CKY. Risk Predictors of Early Childhood Caries Increment—A Systematic Review and Meta-Analysis. J Evid Based Dent Pract. 2022. 10.1016/j.jebdp.2022.101732. 36162891

[13] Li J, Fan W, Zhou Y, Wu L, Liu W, Huang S. The status and associated factors of early childhood caries among 3- to 5-year-old children in Guangdong, Southern China: a provincial cross-sectional survey. BMC Oral Health. 2020. 10.1186/s12903-020-01253-w. PMC7517683 32977784

[14] Razmienė J, Vanagas G, Bendoraitienė EA, Andriuškevičienė V, Slabšinskienė E. Changes in caries prevalence and oral hygiene skills among preschool-aged children in Lithuania between 2000 and 2010. Med (Kaunas). 2012;48:364–70. 23032909

[15] Slabšinskienė E, Milčiuvienė S, Narbutaitė J, Vasiliauskienė I, Andruškevičienė V, Bendoraitienė EA, et al. Severe early childhood caries and behavioral risk factors among 3-year-old children in Lithuania. Medicina. 2010. 10.3390/medicina46020020. 20440088

[16] Zaborskis A, Razmienė J, Razmaitė A, Andruškevičienė V, Narbutaitė J, Bendoraitienė EA, et al. Twelve-Year Changes in Pre-Schoolers’ Oral Health and Parental Involvement in Children’s Dental Care: Results from Two Repeated Cross-Sectional Surveys in Lithuania. Children. 2024. 10.3390/children11111380. PMC11592930 39594955

[17] Stangvaltaite-Mouhat L, Aleksejuniene J, Bendinskaite R, Mdala I, Stankeviciene I, Puriene A, et al. The 20-Year Trends in Caries and Associated Determinants among Adults in Post-Soviet Lithuania: Repeated Cross-Sectional Studies. Caries Res. 2023. 10.1159/000529021. 36626884

[18] Zaborskis A, Kavaliauskienė A, Šidlauskas A. Family Affluence Based Inequality in Oral Health-Related Quality of Life in a Population of Lithuanian Adolescents. Int J Environ Res Public Health. 2019. 10.3390/ijerph16122106. PMC6616952 31197104

[19] Matulaitiene ZK, Zemaitiene M, Zemgulyte S, Milciuviene S. Changes in dental caries and oral hygiene among 7–8 year-old schoolchildren in different regions of Lithuania 1983–2009. Stomatologija. 2012;14:53–9. 23041912

[20] World Health Organization. Oral Health Surveys Basic Methods Fifth Edition. 2013. https://iris.who.int/server/api/core/bitstreams/b00e659f-ab38-4e61-b786-0152d91b5f1b/content. Accessed 20 Jan 2026.

[21] Naing L, Winn T, Rusli BN. Practical Issues in Calculating the Sample Size for Prevalence Studies. Arch Orofac Sci. 2006;1:9–14.

[22] Silness J, Löe H. Periodontal Disease in Pregnancy II. Correlation Between Oral Hygiene and Periodontal Condition. Acta Odontol Scand. 1964. 10.3109/00016356408993968. 14158464

[23] Centre for Quality Assessment in Higher Education. Higher Education. 2025. https://skvc.lrv.lt/en/education-in-lithuania/higher-education/. Accessed 1 May 2026.

[24] James P, Harding M, Beecher T, Browne D, Cronin M, Guiney H, et al. Impact of Reducing Water Fluoride on Dental Caries and Fluorosis. J Dent Res. 2021. 10.1177/0022034520978777. 33345672

[25] Dėl Lietuvos Respublikos sveikatos apsaugos. ministro 2004 m. gruodžio 24 d. įsakymo Nr. V-951 Dėl statistinės apskaitos formos Nr. 027 – 1/a ‘Vaiko sveikatos pažymėjimas’ patvirtinimo pakeitimo. https://www.e-tar.lt/portal/en/legalAct/TAR.BF7FCC3209B0. Accessed 3 Feb 2026.

[26] Maklennan A, Borg-Bartolo R, Wierichs RJ, Esteves-Oliveira M, Campus G. A systematic review and meta-analysis on early-childhood-caries global data. BMC Oral Health. 2024. 10.1186/s12903-024-04605-y. PMC11267837 39049051

[27] Chen KJ, Gao SS, Duangthip D, Lo ECM, Chu CH. Prevalence of early childhood caries among 5-year‐old children: A systematic review. J Investig Clin Dent. 2019. 10.1111/jicd.12376. 30499181

[28] Winkelmann J, Gómez Rossi J, van Ginneken E. Oral health care in Europe: Financing, access and provision. Health Syst Transit. 2022;24(2):1–176. 35833482

[29] Eriksonas L. Lithuania social briefing: Lithuania tightens the regulation of dentists through the legislation changes on state-controlled licensing. 2020. https://china-cee.eu/wp-content/uploads/2020/04/2020s02Lithuania.pdf. Accessed 20 Jan 2026.

[30] National Health Insurance Fund under the Ministry of Health. Reimbursement applies to children’s dental treatment and preventive care. 2022. https://ligoniukasa.lrv.lt/en/news/reimbursement-applies-to-childrens-dental-treatment-and-preventive-care/. Accessed 20 Jan 2026.

[31] Inchley J, Currie D, Budisavljevic S, Torsheim T, Jåstad A, Cosma A, et al. editors. Spotlight on adolescent health and well-being. Findings from the 2017/2018 Health Behaviour in School-aged Children (HBSC) survey in Europe and Canada. International report. Volume 1. Key findings. 2020. https://iris.who.int/server/api/core/bitstreams/a46121d3-8bd4-4267-98ea-c2c7e32c905c/content. Accessed 20 Jan 2026.

[32] Vaičiūnas T, Golambiauskas V, Abdrakhmanova S, Melkumova M, Movsesyan E, Sturua L, et al. Oral hygiene predicts lower life satisfaction and subjective health: experience of post-Soviet countries. Eur J Pediatr. 2024. 10.1007/s00431-024-05743-9. PMC11527959 39425767

[33] Eurostat. Preventive health care expenditure statistics. 2025. https://ec.europa.eu/eurostat/statistics-explained/index.php?title=Preventive_health_care_expenditure_statistics. Accessed 21 Jan 2026.

[34] Eurostat. Income inequality across Europe in 2021. 2023. https://ec.europa.eu/eurostat/web/products-eurostat-news/w/ddn-20230113-1. Accessed 20 Jan 2026.

[35] OECD/European Commission. Health at a Glance: Europe 2024: State of Health in the EU Cycle. 2024. 10.1787/b3704e14-en. Accessed 20 Jan 2026.

[36] Knorst JK, Sfreddo CS, de Meira F, Zanatta G, Vettore FB, Ardenghi MV. Socioeconomic status and oral health-related quality of life: A systematic review and meta‐analysis. Community Dent Oral Epidemiol. 2021. 10.1111/cdoe.12616. 33368600

[37] Rouxel P, Chandola T. Socioeconomic and ethnic inequalities in oral health among children and adolescents living in England, Wales and Northern Ireland. Community Dent Oral Epidemiol. 2018. 10.1111/cdoe.12390. PMC6849874 29888400

[38] Kamińska A, Szalewski L, Batkowska J, Wallner J, Wallner E, Szabelska A, et al. The dependence of dental caries on oral hygiene habits in preschool children from urban and rural areas in Poland. Ann Agric Environ Med. 2016. 10.5604/12321966.1226863. 28030940

[39] Stasch N, Ganahl K, Geiger H. Soziale Ungleichheit in der Zahngesundheit von Kindern. Prävention und Gesundheitsförderung. 2023. 10.1007/s11553-021-00929-7.

[40] United Nations Department of Economic and Social Affairs. The Sustainable Development Goals Report 2025. 2025. https://unstats.un.org/sdgs/report/2025/The-Sustainable-Development-Goals-Report-2025.pdf. Accessed 20 Jan 2026.

[41] Foláyan ḾOluwátóyìn, de Barros Coelho EMR, Feldens CA, Gaffar B, Virtanen JI, Kemoli A, et al. A scoping review on the associations between early childhood caries and sustainable cities and communities using the sustainable development goal 11 framework. BMC Oral Health. 2024. 10.1186/s12903-024-04521-1. PMC11214204 38943110

[42] Metreau E, Young KE, Eapen SG. World Bank country classifications by income level for 2024–2025. 2024. https://blogs.worldbank.org/en/opendata/world-bank-country-classifications-by-income-level-for-2024-2025. Accessed 21 Jan 2026.

[43] Ubarevičienė R, van Ham M. Population decline in Lithuania: who lives in declining regions and who leaves? Regional Studies. Reg Sci. 2017. 10.1080/21681376.2017.1313127.

[44] OECD. Reviews of National Policies for Education. Education in Lithuania. 2017. 10.1787/9789264281486-en. Accessed 1 May 2026.

[45] OECD. OECD Reviews of Labour Market and Social Policies: Lithuania. 2018. 10.1787/9789264189935-en. Accessed 20 Jan 2026.

[46] State Data Agency. Statistics Lithuania. Lithuania in figures (edition 2023). 2023. https://osp.stat.gov.lt/namu-ukiu-pajamos-ir-gyvenimo-salygos. Accessed 20 Jan 2026.

[47] Chartier O, Salle E, Kull M, Miller D, Nieto E, Vestergård L, Potters J, Slätmo E, Zomer B, Iadecola F. Long-Term Vision for Rural Areas: Contribution from SHERPA science-society-policy platforms. 2021. 10.5281/zenodo.4557440. Accessed 20 Jan 2026.

[48] Lithuanian Health Information Centre. Health Statistics of Lithuania. 2006. https://www.hi.lt/uploads/Statistikos_leidiniai_Sveikatos_statistika/la2006.pdf. Accessed 20 Jan 2026.

[49] Janulyte V, Aleksejuniene J, Puriene A, Peciuliene V, Benzian H. Current employment characteristics and career intentions of Lithuanian dentists. Hum Resour Health. 2014. 10.1186/1478-4491-12-74. PMC4290105 25528155

[50] Crouch E, Nelson J, Merrell MA, Martin A. The oral health status of America’s rural children: An opportunity for policy change. J Public Health Dent. 2021. 10.1111/jphd.12444. 33501720

[51] Northridge ME, Kumar A, Kaur R. Disparities in Access to Oral Health Care. Annu Rev Public Health. 2020. 10.1146/annurev-publhealth-040119-094318. PMC7125002 31900100

[52] Lou Y, Sun Z, Ma H, Cao D, Sun M, Wang Q, et al. Odontogenic infections in the antibiotic era: approach to diagnosis, management, and prevention. Infection. 2024. 10.1007/s15010-023-02117-5. 37926767

[53] Teal L, Sheller B, Susarla HK. Pediatric Odontogenic Infections. Oral Maxillofac Surg Clin North Am. 2024. 10.1016/j.coms.2024.03.005. 38777729

[54] Harris JC, Balmer RC, Sidebotham PD. British Society of Paediatric Dentistry: a policy document on dental neglect in children. Int J Paediatr Dent. 2018. 10.1111/j.1365-263X.2009.00996.x. 19470009

[55] Khalid G, Metzner F, Pawils S. Prevalence of dental neglect and associated risk factors in children and adolescents—A systematic review. Int J Paediatr Dent. 2022. 10.1111/ipd.12923. 34585811

[56] Chen L, Hong J, Xiong D, Zhang L, Li Y, Huang S, et al. Are parents’ education levels associated with either their oral health knowledge or their children’s oral health behaviors? A survey of 8446 families in Wuhan. BMC Oral Health. 2020. 10.1186/s12903-020-01186-4. PMC7353758 32652985

[57] Folayan MO, Coelho EMR, de Ayouni B, Nguweneza I, Al-Batayneh A, Daryanavard OB. Association between early childhood caries and parental education and the link to the sustainable development goal 4: a scoping review. BMC Oral Health. 2024. 10.1186/s12903-024-04291-w. PMC11064360 38698356

[58] Minervini G, Franco R, Marrapodi MM, Di Blasio M, Ronsivalle V, Cicciù M. Children oral health and parents education status: a cross sectional study. BMC Oral Health. 2023. 10.1186/s12903-023-03424-x. PMC10594879 37875845

[59] Plutzer K, Spencer AJ. Efficacy of an oral health promotion intervention in the prevention of early childhood caries. Community Dent Oral Epidemiol. 2008. 10.1111/j.1600-0528.2007.00414.x. 19145720

[60] Hajishafiee M, Kapellas K, Listl S, Pattamatta M, Gkekas A, Moynihan P. Effect of sugar-sweetened beverage taxation on sugars intake and dental caries: an umbrella review of a global perspective. BMC Public Health. 2023. 10.1186/s12889-023-15884-5. PMC10224604 37237341

[61] Ministry of Finance of the Republic of Lithuania. Excise Duties. 2026. https://finmin.lrv.lt/en/competence-areas/taxation/main-taxes/excise-duties/. Accessed 21 Jan 2026.

