# Supplementary material for: Residence- and parental education-related social inequalities in early childhood caries: cross-sectional national Lithuanian oral health survey
Source: BMC Oral Health. 2026 Jul 18;26:2138. doi: 10.1186/s12903-026-09311-5 (PMC13721721; doi:10.1186/s12903-026-09311-5)
Supplement: Supplementary file 1 [file 12903_2026_9311_MOESM1_ESM.docx]

**Additional file 1.** Sensitivity analysis for multivariable negative binomial regression of the association between place of residence, parental education and early childhood caries experience (d_3_mft index and its individual components d_3_t, mt, ft) in participants (n=431).

| DETERMINANTS | **d_3_mft (decayed, missing, filled teeth)** | | **d_3_t (decayed teeth)** | | **mt (missing teeth)** | | **ft (filled teeth)** | |
| --- | --- | --- | --- | --- | --- | --- | --- | --- |
|  | RM (95% CI) | P value | RM (95% CI) | P value | RM (95% CI) | P value | RM (95% CI) | P value |
| **Sex** (ref. Male) | | | | | | | | |
| Female | 1.16  (0.92; 1.46) | 0.218 | 1.26  (0.97; 1.63) | 0.078 | 1.03  (0.58; 1.84) | 0.919 | 1.06  (0.80; 1.41) | 0.687 |
| **Place of residence** (ref. Urban) | | | | | | | | |
| Peri-urban | 1.49  (1.09; 2.02) | 0.011 | 1.66  (1.19; 2.33) | 0.003 | 1.16  (0.58; 2.33) | 0.667 | 1.31  (0.91; 1.88) | 0.141 |
| Rural | 1.46  (1.02; 2.11) | 0.040 | 1.91  (1.30; 2.81) | 0.001 | 0.62  (0.25; 1.58) | 0.319 | 0.91  (0.57; 1.45) | 0.691 |
| **Parental education** (ref. University) | | | | | | | | |
| College | 1.32  (1.00; 1.75) | 0.051 | 1.31  (0.96; 1.79) | 0.092 | 2.54  (1.25; 5.16) | 0.010 | 1.20  (0.86; 1.67) | 0.275 |
| Secondary or lower | 1.04  (0.77; 1.41) | 0.797 | 1.27  (0.92; 1.77) | 0.151 | 2.48  (1.20; 5.16) | 0.015 | 0.58  (0.40; 0.86) | 0.006 |
| **Fluoride in community water** (ref. ≥0.7 mg/L) | | | | | | | | |
| <0.7 mg/L | 1.40  (1.06; 1.84) | 0.016 | 1.33  (0.98; 1.80) | 0.066 | 1.84  (0.88; 3.84) | 0.103 | 1.50  (1.07; 2.10) | 0.019 |
| **Oral hygiene** (ref. Good) | | | | | | | | |
| Moderate | 1.85  (1.45; 2.36) | <0.001 | 2.29  (1.75; 2.99) | <0.001 | 0.55  (0.27; 1.13) | 0.103 | 1.53  (1.14; 2.06) | 0.005 |
| Poor | 3.93  (2.51; 6.16) | <0.001 | 4.90  (3.04; 7.87) | <0.001 | 3.03  (1.26; 7.31) | 0.013 | 2.38  (1.38; 4.10) | 0.002 |
